# Interferon gamma release assays for diagnosis of osteoarticular tuberculosis: A systematic review and meta-analysis

**DOI:** 10.1371/journal.pone.0269234

**Published:** 2022-06-30

**Authors:** Chunnian Ren, Jie Tang, Liangfeng Xia

**Affiliations:** 1 Department of Cardiothoracic Surgery, Chengdu Women’s and Children’s Central Hospital, School of Medicine, University of Electronic Science and Technology of China, Chengdu, China; 2 Department of Biostatistics and Epidemiology, School of Public Health, Shenyang Medical College, Shenyang, China; Food and Drug Administration, UNITED STATES

## Abstract

**Background:**

Although the Interferon Gamma Release Assays (IGRA) is often used to identify latent tuberculosis, it also plays a crucial role in diagnosing active extrapulmonary tuberculosis. Some studies have assessed the use of IGRA as a biomarker for osteoarticular tuberculosis (OATB), which is elevated following TB infection. Still, conclusive results about its effectiveness have not been reported.

**Method:**

We searched PubMed, Embase, and Cochran databases. We obtained literature related to the diagnosis of OATB by IGRA, and the retrieval period was from the establishment of the database to June 2021. The bivariate random effect model was used to summarize the sensitivity, specificity, and accuracy of other indicators in diagnosing OATB by IGRA, and the forest plot and receiver operating characteristic (ROC) curve were used for testing.

**Results:**

We included seven studies involving 643 subjects in diagnosing OATB by IGRA. The comprehensive sensitivity and specificity were 0.84 (95% CI, 0.70–0.92) and 0.78 (95% CI, 0.66–0.87), respectively. The area under the curve (AUC) was 0.87.

**Conclusion:**

In blood samples, the diagnostic accuracy of IGRAS is poor in patients with suspected OAT. We conclude that IGRA may not be appropriate for patients with OATB.

## Introduction

On a global scale, tuberculosis is an infectious disease with high morbidity and mortality, causing two to three million deaths annually and presenting approximately nine million new cases [1, 2]. Extrapulmonary tuberculosis accounted for 1 in 8 of all tuberculosis, of which the incidence of osteoarticular tuberculosis (OATB) was 11.3%-34.5% [3, 4]. The early symptoms of OATB are not obvious, and the signs and imaging features are not typical, leading to difficulties in diagnosis[5, 6]. At present, the diagnosis of OATB is based on the pathogeny or pathology of surgical (biopsy) specimens. However, due to limited medical conditions or unwillingness of patients, or inability to undergo invasive examination, the diagnosis becomes a dilemma, leading to misdiagnosis and missed diagnosis. Besides, traditional methods of culturing tuberculosis bacteria take weeks to obtain positive results and have a low detection rate [7]. The incidence of OATB with atypical clinical manifestations is increasing [8]. Early diagnosis of OATB is essential for controlling this infectious disease and further treatment. Nowadays, the number of studies on the diagnosis of OATB with Interferon Gamma Release Assays (IGRA) is climbing each year. However, due to the small sample size of individual studies, differences in study design, and source of patients, the accuracy of IGRA varies, and there is no evidence-based medicine evidence on its clinical diagnostic value.

The tuberculin skin test (TST), which uses tuberculin pure protein derivatives as diagnostic reagents, can trigger specific skin allergic reactions to organisms that have been infected with tuberculosis or have been vaccinated with BCG. TST failed to be widely deployed because, for one thing, the population inoculated with BCG-vaccinated, and the people in high-incidence countries may be tested positive, and it has poor specificity. In recent years, interferon release assay (IGRAs) has been used as an immune diagnostic tool for tuberculosis. IGRA is an immune-based blood detection method that can quantify the interferon-γ released by T cells under the stimulation of two mtb-specific antigens (culture filter protein (CFP) -10) and early secretory antigen target (ESAT) -6) [9–11]. In contrast to TST, IGRA did not cross-react with BCG strains and most nontuberculous mycobacteria (NTM) species [12]. QuantiFERON-TB Gold In-Tube test (QFT-GIT; QIAGEN, Hilden, Germany) and Enzyme-linked immunosorbent SPOT (ELISA) assay (T-SPOT.TB; Oxford Immunotec Limited, United Kingdom) are two of the most common testing methods for IGRAs in the market. QFT-GIT is a test that uses a mixture of Mycobacterium tuberculosis-specific antigen (ESAT-6 and CFP-10) to stimulate specific T-cells in peripheral blood in vitro to detect the amount of IFN-γ released. T-SPOT Tuberculosis is an enzyme-linked immunoblot assay that measures the number of cells in the peripheral blood that respond to ESAT-6 and CFP-10 antigens and produce IFN-Y [13, 14]. Although IGRAs can not distinguish active from latent tuberculosis, they use a blood or humoral samples to diagnose pulmonary and extrapulmonary tuberculosis [15, 16]. IGRAs are simple and noninvasive in a quicker diagnosis of OATB. We conducted this meta-analysis to obtain more critical evidence about the diagnostic accuracy of IGRA in diagnosing OATB.

## Materials and methods

The methodology followed for the systematic review is the Preferred Reporting Items for Systematic Reviews and Meta-Analyses (PRISMA) [17]. We evaluated the quality of studies with the Quality Assessment of Diagnostic Accuracy Studies (QUADAS) checklist [18].

### Search strategy

We systematically searched all published studies in English in electronic databases PubMed, Embase, and Cochrane Library from 1999 through Jun 2021. The search strategy terms included as follows: (interferon-gamma OR interferon-gamma release assay OR gamma-interferon OR IFN interferon-gamma OR interferon OR IGRA OR interferon release assay OR interferon-gamma assay OR T-SPOT OR enzyme-linked immunosorbent spot OR ELISpot OR QuantiFERON OR T cell response OR T cell-based assay) AND (Tuberculosis, Bone OR Tuberculoses, Bone OR Tuberculoses, Osteoarticular OR Bone Tuberculosis OR Bone Tuberculoses OR Osteoarticular Tuberculosis OR Osteoarticular Tuberculosis OR Joint Tuberculosis OR Joint Tuberculoses OR Tuberculoses, Joint OR Tuberculosis, Joint OR Tuberculosis, Osteoarticular). We also identified additional studies from the references in these papers selected.

### Study selection

**Fig 1:**
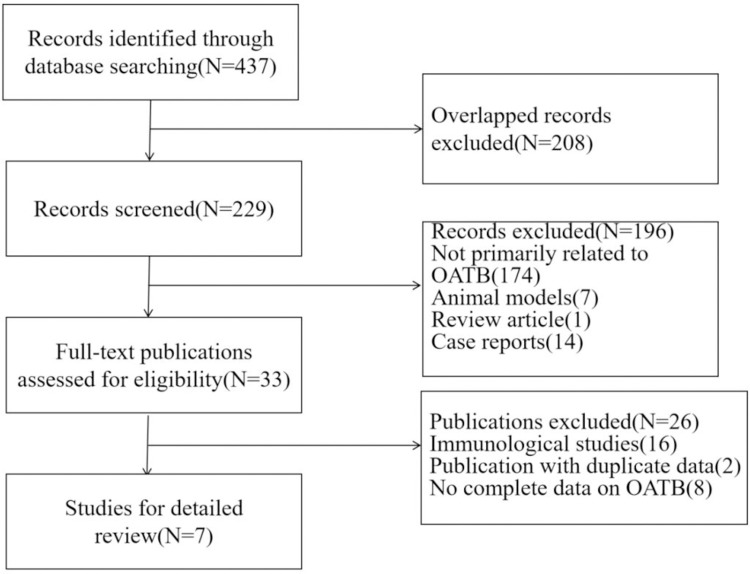
Flow chart of the study selection process. The flowchart shows the procedure used to select the articles for the qualitative and quantitative synthesis.

We do a preliminary reading of titles and abstracts to filter out articles that might be suitable for a more detailed evaluation (Fig 1). Two reviewers (Jie Tang and Liangfeng Xia) independently conducted a second review of the full text of all the selected articles to determine suitable studies for inclusion. If there are differences, resolve them by consensus. Inclusion criteria (1) Original data, including true negative value (TN), true positive value (TP), false negative value (FN), and a false positive value (FP) of IGRA in peripheral blood were obtained directly or indirectly in the diagnosis of OATB; (2) Patients in the experimental group were diagnosed by etiology, pathology or clinical diagnosis, or after anti-tuberculosis treatment, symptoms were significantly relieved, and various auxiliary examinations tended to be expected; Patients in the control group included diseases that needed to be differentiated from bone and joint tuberculosis during a clinical or pathological examination, including nonspecific infection and tumor; (3)The number of cases of bone and common tuberculosis was more than 10, and the difference was statistically significant compared with the control group. Exclusion criteria (1) literature and data repeatedly published in each database; (2) it is not possible to extract values for diagnostic accuracy or allow sensitivity and specificity to be calculated from observations reported by numerical data or dot plots; (3) Meeting abstract, case report, and review.

The Diagnostic reference standard for OATB is (1) positive culture or smear of acid-fast bacilli (AFB), (2) histopathological evidence of tuberculous granuloma (proliferative or caseous necrosis), and (3) radiological features consistent with ultrasound or CT scan of the osteoarthrosis. Patients are classified as probable to have tuberculosis if they respond well to anti-tuberculosis treatment and have radiographic features consistent with OATB.

### Data extraction

According to the preset inclusion and exclusion criteria, two reviewers (Jie Tang and Liangfeng Xia) independently evaluated the full texts for eligibility. The following details were extracted from all included studies: first author, year of publication, country of analysis, sample size, the age range of participants, the proportion of HIV infection, IGRA method, number of positive, negative, and non-conclusive results. Indeterminate results may occur in IGRA tests, excluding these patients may change the sensitivity and specificity. They were excluded before specificity and included as false negatives when calculated sensitivity [15, 19].

### Quality assessment

The quality of these studies was evaluated by diagnostic accuracy research quality assessment-2 (QUADAS-2). We evaluated the risk of bias and applicability of the study. The risk of bias is mainly divided into four parts, including case selection, diagnostic gold standard, indicator test, flow, and timing. At the same time, we conduct an applicability assessment on the first three fields. Each field is classified as high, low, and unknown, corresponding to 0, 1, and 0. An overall score greater than or equal to 4 is considered high quality. Any differences are resolved by consensus between the two reviews [20].

### Statistical analysis

The sensitivity and specificity of each study were collected and calculated, and the corresponding 95% confidence interval (95% CI) was given. The results of sensitivity, specificity, positive likelihood ratio (PLR), negative likelihood ratio (NLR), and diagnostic advantage ratio (DOR) were summarized using the bivariate random effect model. The receiver operating characteristic curve (ROC) is the coordinate graph composed of a false-positive rate on the horizontal axis and a valid positive rate on the vertical axis. The curve is drawn by the different results obtained by the receiver under specific stimulus conditions using different judgment criteria. Summarize the receiver operating characteristic (SROC): The meta-analysis of multiple different trials with the same indicator can be expressed by a ROC curve named SROC according to the weight of their odds ratio (OR). We can obtain the specificity and sensitivity of this group of studies [21]. The I2 test assessed heterogeneity and I^2^ was more than 25%, 50%, and 75%, defined as low, moderate, and high, respectively [22]. By Spearman rank correlation, P < 0.05 was the significant threshold. Assess potential publication bias using funnel plots (Deeks method). All statistical tests were analyzed using Stata (intercooler version 14.0; Stata Corp., College Station, TX).

## Results

### Study characteristics

We searched 437 articles and eventually included 7 studies that met our criteria (Table 1) [1, 23–28]. These seven studies were conducted between 2009 and 2020, six of which were in China (589 patients) and one in South Korea (54 patients). The IGRA method used in all studies was the T-SPOT.TB (Oxford Immunology Technologies, Abington, UK).

**Table 1:** Summary characteristics of studies included in the meta-analysis.

authors,year(reference)	Country	OATB/non-OATB patients^a^	Age (yr)^b^		Gender (M: F)		HIV status of OATB patients	IGRA method	Test results						
									OATB patients			non-OATB patients			
			OATB patients	non-OATB patients					TP	FN	I		TN	FP	I
					OATB patients	non-OATB patients									
Cho 2010 [23]	Korea	23(23)/32	52.3±15.5	58.8 ± 16.6	11/12	14/18	All negative	T-SPOT.TB	22	0	1		18	13	0
Jia 2013 [24]	China	86(86)/24	40.5(18–76)	45.5 (16–80)	50/36	15/9	All negative	T-SPOT.TB	81	5	0		17	7	0
Li 2020 [25]	China	92(35)/137	53±17	55 ±18	54/38	85/52	All negative	T-SPOT.TB	67	25	0		94	43	0
Liao 2009 [26]	Taiwan	15(9)/3	NR	NR	NR	NR	NR	T-SPOT.TB	12	3	0		3	0	0
Tang 2016 [1]	China	86(86)/30	36±14	37±9	49/37	13/17	All negative	T-SPOT.TB	70	16	0		29	1	0
WU 2014 [27]	China	90(29)/54	43.5±16.5	43.5 ± 17.6	46/44	34/20	NR	T-SPOT.TB	10	12	0		17	4	0
Zhou 2019 [28]	China	39(12)/34	NR	55.0±20.0	23/28	22/13	All negative	T-SPOT.TB	35	4	0		28	6	0

^a^Figures in parentheses are the numbers of patients with definite tuberculosis (diagnosis confirmed by microbiological and/or histopathological investigations).

^b^Figures are means±SD or medians with age ranges.

M, male; F, female; IGRA, interferon-gamma release assay; TP, true positive; FN, false negative; I, indeterminate; TN, true opposite; FP, false positive. NR, not reported in study; OATB, osteoarticular tuberculosis.

### Quality of studies included

All studies prospectively or retrospectively included suspected tuberculosis patients. Three studies excluded patients with an uncertain diagnosis, which may exaggerate the diagnostic accuracy of the index test, so the risk of bias was higher [1, 23, 24]. In addition, a study excluded patients without IGRA results and had an increased risk of selection bias [25]. In the only study, researchers adopted the blind method to diagnose patients in the laboratory [23], while other studies did not report the use of the blind approach. In addition to two studies, all studies used manufacturer-specified thresholds to classify the results. One uses the point noted in the previous literature, which is not far from the threshold recommended by the manufacturer [26]. Another study did not mention the use of threshold in the article [27]. Only one study reported uncertainty in the test, but We excluded the uncertainty from the analysis [23]. In addition to one study, all studies provided detailed information on the reference standard for diagnosing OATB [25]. Three studies have shown that each OATB patient has clear pulmonary tuberculosis [1, 23, 24]. Some patients had similar definite diagnoses [1, 25–27].

**Fig 2:**
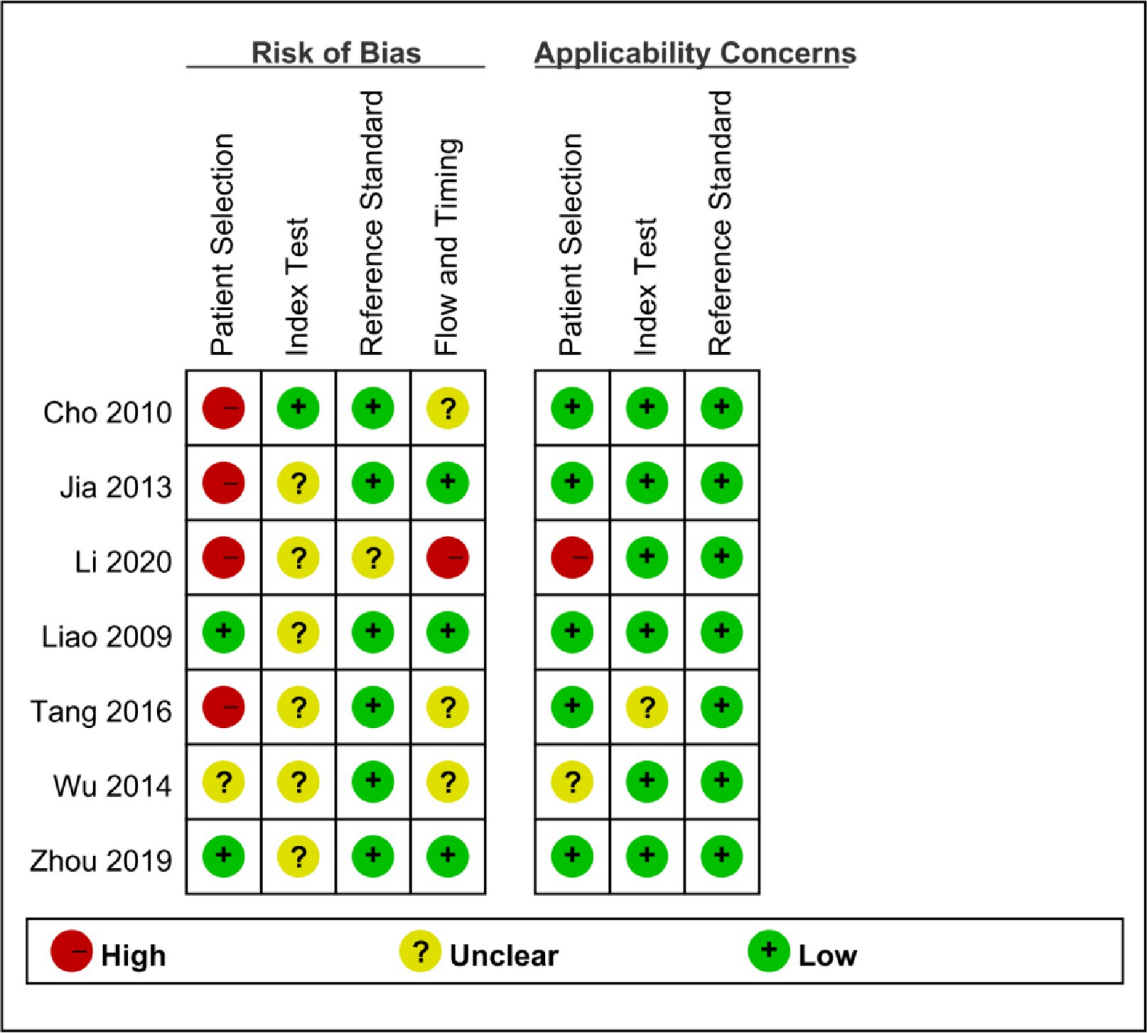
Risk of bias assessment of the included studies. According to the QUADAS-2 (Quality Assessment of Diagnostic Accuracy Studies-2), the summary of the methodological quality of studies.

The quality of seven studies was listed, showing that only two studies had a low bias risk (Fig 2). Four studies with patient selection bias were classified as high-risk studies without clear diagnostic criteria or excluding patients with uncertain clinical diagnoses. Only one study has a low-risk bias on the index test because it is explained without understanding other tests. Only one study showed a high risk of applicability.

### Diagnostic performance

**Fig 3:**
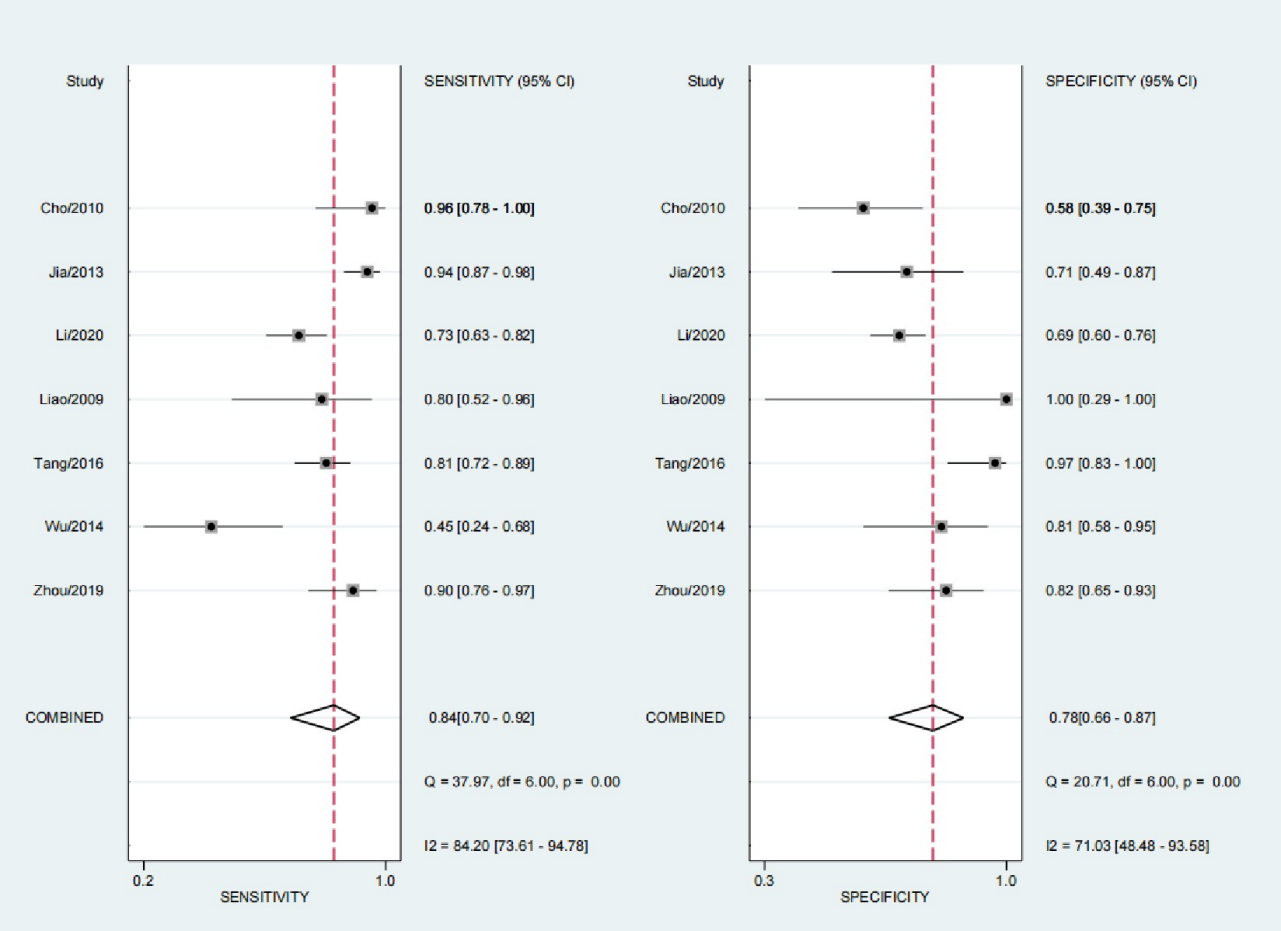
Evaluation of the sensitivity and specificity of interferon-γ release assay in the diagnosis of OATB: A study forest plot. Filled squares represent sensitivity/specificity estimates from studies done using. interferon-γ release assay. See references 24 to 30 for details. Thresholds for the interpretation of I2 are as follows: 0% to 40%: might not be essential; 30% to 60%: may represent moderate heterogeneity; 50% to 90%: may represent substantial heterogeneity; 75% to 100%: considerable heterogeneity.

**Fig 4:**
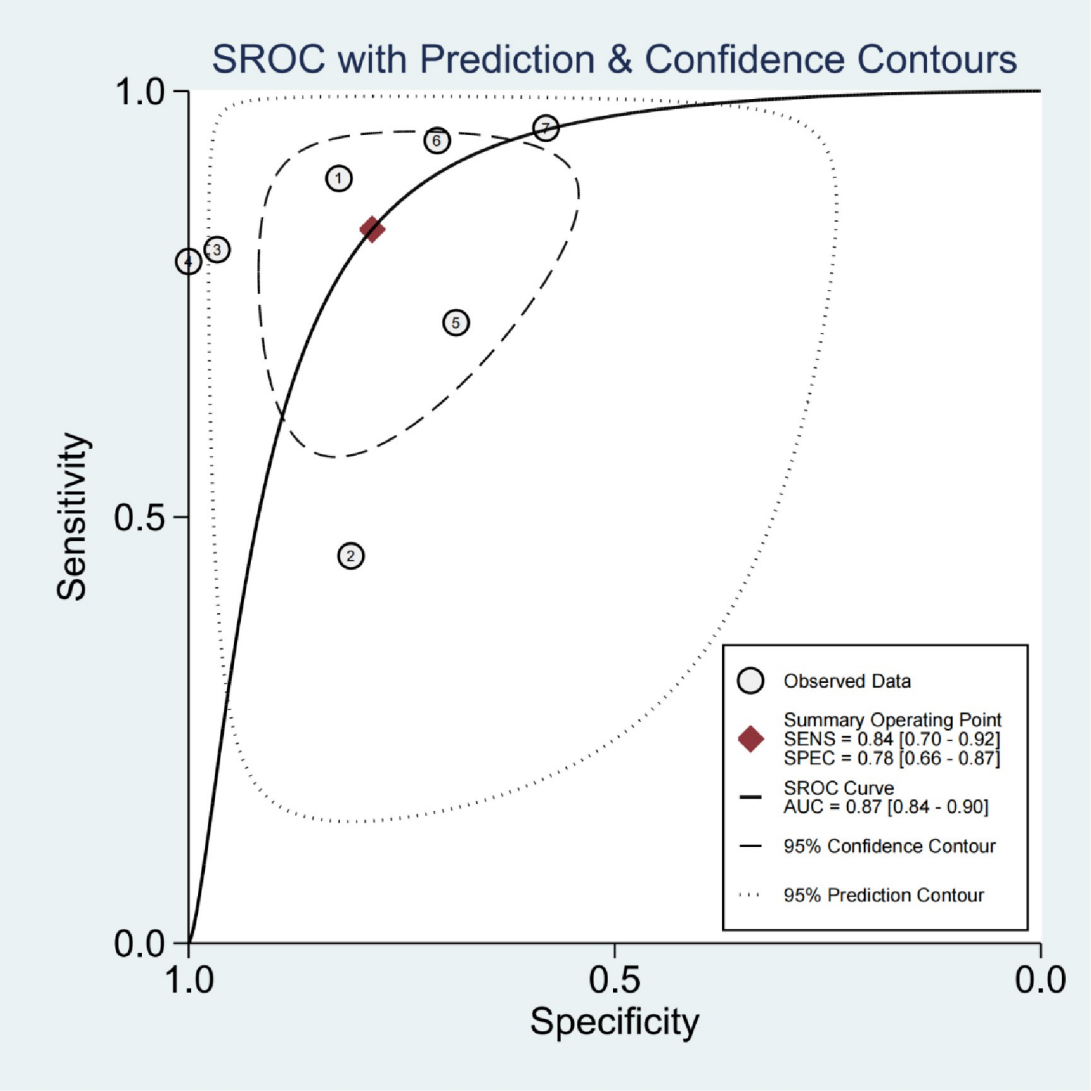
Summarize the receiver operating characteristic (SROC) curve and summarize the performance of interferon-gamma release assay in the diagnosis of OATB. Each study is represented by an open circle whose size is proportional to the inverse standard error of sensitivity and specificity. The filled square represents the summary estimate of the test accuracy, with the surrounding dashed zone outline denoting the 95% confidence region around this estimate.

The sensitivity range of all studies was 0.45 to 0.96, and the total estimation was 0.84 (95% CI, 0.70 to 0.92). The specificity range of all studies was 0.58–1.0, and the comprehensive estimation was 0.78 (95% CI, 0.66–0.78) (Fig 3). The combined estimates of PLR, NLR and DOR were 3.9 (95% CI, 2.4–6.4), 0.21 (95% CI, 0.11–0.39) and 19 (95% CI, 8–46), respectively. The corresponding ROC curve is shown in Fig 4. The position and shape of the curve indicate that the total sensitivity/specificity is a suboptimal discriminator. I^2^ of sensitivity and specificity was 84.20% and 71.03%, respectively, with moderate and severe heterogeneity between studies.

In subgroup analysis, only the study included patients with immunosuppressive conditions with lesser heterogeneity insensitivity. However, those not excluded patients with an uncertain diagnosis, patients with or without previous tuberculosis infection history, patients with the immunosuppressive condition, and those included with patients with other diseases were associated with lesser heterogeneity in specificity (Table 2). Nevertheless, estimated variations in pooled specificity and sensitivity between subgroups were slight. The threshold effect of statistical results showed no significance, and the Spearman correlation coefficient was 0.28 (P = 0.54).

**Table 2:** Subgroup analysis for exploration of factors influencing heterogeneity.

Parameter	Category (no. of studies)	Pooled sensitivity (95% CI)	*I*2 (%)	Pooled specificity (95% CI)	*I*2 (%)	Pooled diagnostic odds ratio (95% CI)
Exclude patients with uncertain diagnosis	Excluded(3)	0.89(0.83–0.93)	76.2	0.75(0.65–0.84)	86.8	48.10(18.41–125.67)
	Not excluded(4)	0.74(0.67–0.80)	79.2	0.73(0.66–0.79)	46.0	9.81(3.23–29.81)
Total study sample	>100 patients (3)	0.83(0.78–0.87)	87.3	0.73(0.66–0.79)	85.1	25.65(4.06–162.25)
	<100 patients(4)	0.80(0.71–0.87)	85.2	0.74(0.64–0.83)	59.5	16.37(4.09–65.48)
Previous TB infection history	TB infection history(3)	0.85(0.79–0.89)	89.4	0.67(0.60–0.74)	0.0	16.49(3.79–71.78)
	Not TB infection history(4)	0.78(0.71–0.85)	80.4	0.88(0.79–0.94)	44.5	23.84(4.36–130.32)
Immunosuppressive condition	Included(3)	0.93(0.87–0.97)	36.6	0.66(0.52–0.78)	44.2	35.36(12.66–98.77)
	Not included(4)	0.76(0.70–0.81)	81.5	0.76(0.70–0.81)	79.9	15.12(3.73–61.24)
Patients With other diseases	Included(4)	0.84(0.79–0.89)	84.2	0.68(0.61–0.74)	20.3	16.94(4.71–60.89)
	Not included(3)	0.78(0.71–0.85)	86.9	0.87(0.78–0.93)	56.4	24.09(3.08–188.54)

**Fig 5:**
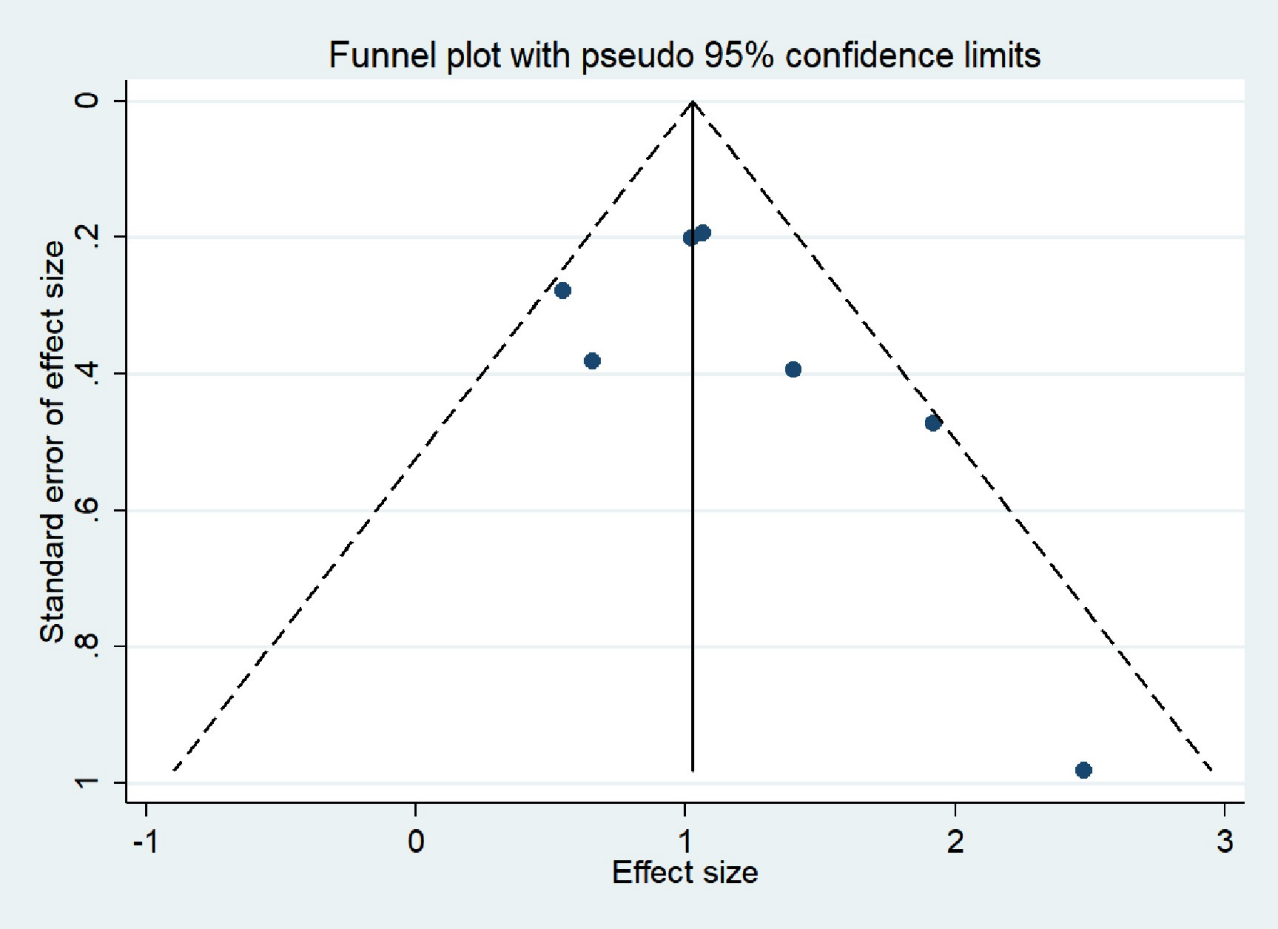
Deeks’ funnel plots for publication bias. Deeks funnel plot assessment test evaluates the potential publication bias for interferon-gamma release assays on OATB. The plot shows the symmetric distribution of the log of diagnostic odds ratios against the inverse root of effective sample sizes (ESS), indicating the absence of any publication bias.

A Deeks’ funnel plot was symmetric and showed a lack of publication bias (P = 0.50) (Fig 5). There was no evidence of publication bias in our evaluation.

## Discussion

As an in vitro immunodiagnostic test, IGRA is an excellent diagnostic tool for LTB and is increasingly being used to detect Mycobacterium tuberculosis infection [29]. This emerging technology is applied not only to latent tuberculosis but also to examine the diagnostic value of IGRA for extrapulmonary tuberculosis based on different sites of infection [30]. There has been no meta-analysis of the role of IGRA in the diagnosis of OATB. Therefore, we conducted this meta-analysis to comprehensively evaluate the overall diagnostic accuracy of IGRA for OATB. The Pooled sensitivity and specificity estimates of IGRA were 0.84 and 0.78, respectively. The pooled area under the ROC curve (AUC) was 0.87, indicating poor diagnostic accuracy. AUC is a comprehensive indicator of acute sensitivity and specificity. An AUC greater than 0.9 is generally considered as high accuracy in diagnostic tests [30]. Meta-analysis is a collection of the results of previous individual studies. Still, the heterogeneity of the combined studies is high, and no apparent cause of heterogeneity is found through subgroup analysis. More high-quality studies are required to obtain more reliable results. Several possible factors contribute to the high heterogeneity. Some non-OATB patients may have latent tuberculosis status because some studies included patients previously suffering from tuberculosis, and the false positives caused by them may reduce the specificity. Different sites of OATB may generate different amounts of interferon-gamma release; the various affected areas of included studies may cause heterogeneity. If subgroup analysis of different places is available, We may obtain more accurate results. Besides, only one study showed inconclusive results from the IGRA, and other studies were not clear; blind exclusion may result in inaccurate specificity. Most of the studies included in this meta-analysis are Chinese studies, and only one Korean study, which cannot analyze for country differences. In general, the results of this meta-analysis suggested that the diagnostic accuracy of IGRA in OATB was poor; It was also mentioned in other systematic reviews [16, 31]. This study focused on osteoarticular tuberculosis and found similar results as other tuberculosis.

There are some limitations to our meta-analysis. Because the quality of some studies was poor, the selection of patients was not fully randomized, and patient screening conditions were inconsistent. The small number of patients included in some studies also contributed to the inaccurate results. Although most studies used OATB diagnostic reference standards, the diagnostic criteria of some studies were not perfect. Because in the clinic, it is impossible to diagnose thoroughly by microbiological/pathological. The majority of patients included in this study were immunocompetent, and patients included in other studies included immunocompromised, which contributed to the heterogeneity. Sensitivity and specificity, as we know, can be influenced by the prevalence of the disease. The studies included in this study were all based in Asia. Therefore, our conclusions may only be applicable apply to the Asian region.

## Conclusion

To sum up, IGRA(whole blood) exhibits poor diagnostic accuracy in OATB. Although IGRA is a noninvasive diagnostic method for OATB, its poor diagnostic accuracy does not benefit the diagnosis. We suggest that it should not be used as a standard diagnostic tool.

## Supporting information

S1 Checklist (DOCX)

S1 Dataset (XLSX)
